# Multiple Antioxidative and Bioactive Molecules of Oats (*Avena sativa* L.) in Human Health

**DOI:** 10.3390/antiox10091454

**Published:** 2021-09-13

**Authors:** Il-Sup Kim, Cher-Won Hwang, Woong-Suk Yang, Cheorl-Ho Kim

**Affiliations:** 1 Advanced Bio-Resource Research Center, Kyungpook National University, Daegu 41566, Korea; 92kis@hanmail.net; 2 Global Leadership School, Handong Global University, Pohang 37554, Gyeongsangbuk-Do, Korea; 3 Nodaji Co., Ltd., Pohang 37927, Gyeongsangbuk-Do, Korea; 4 Department of Biological Sciences, SungKyunKwan University, Suwon 16419, Gyunggi-Do, Korea; 5 Samsung Advanced Institute of Health Science and Technology (SAIHST), Sungkyunkwan University, Seoul 06351, Korea

**Keywords:** oat, *Avena sativa* L., β-glucan, avenanthramide, functionality, health benefit

## Abstract

Oats (*Avena sativa* L.) are rich in protein, fiber, calcium, vitamins (B, C, E, and K), amino acids, and antioxidants (beta-carotene, polyphenols, chlorophyll, and flavonoids). β-glucan and avenanthramides improve the immune system, eliminate harmful substances from the body, reduce blood cholesterol, and help with dietary weight loss by enhancing the lipid profile and breaking down fat in the body. β-glucan regulates insulin secretion, preventing diabetes. Progladins also lower cholesterol levels, suppress the accumulation of triglycerides, reduce blood sugar levels, suppress inflammation, and improve skin health. Saponin-based avanacosidase and functional substances of flavone glycoside improve the immune function, control inflammation, and prevent infiltration in the skin. Moreover, lignin and phytoestrogen prevent hormone-related cancer and improve the quality of life of postmenopausal women. Sprouted oats are rich in saponarin in detoxifying the liver. The literatures have been reviewed and the recent concepts and prospects have been summarized with figures and tables. This review discusses recent trends in research on the functionality of oats rather than their nutritional value with individual immunity for self-medication. The oat and its acting components have been revisited for the future prospect and development of human healthy and functional sources.

## 1. Introduction

So-called “superfoods” are food products that boost the immunity of the body through their rich contents of nutrients and antioxidants. Representative examples of superfoods include tomatoes, spinach, broccoli, salmon, garlic, and blueberries [1]. Recently, interest in so-called “functional grains”, which are also rich in nutrients and antioxidants, has increased, particularly in the United States of America, Europe, and Japan [2]. These functional grains include oats, quinoa, lentils, chickpeas, amaranth, chia seeds, wild rice, and flaxseeds. Although there are differences in the level of nutrients between each grain, these grains are rich in protein, vitamins, minerals, and dietary fiber compared to the commonly consumed rice (white rice), barley, and wheat. Functional grains help the modern population suffering from “poverty in the midst of plenty” due to busy daily life, irregular meals, and frequent consumption of take-out restaurant meals to ingest abundant nutrients [3,4,5,6,7,8].

Oats (*Avena sativa* L.) are biennial herbaceous plants that belong to the Poaceae family. They are one of the representative crops that grow in cool and humid weather conditions [9]. Oats have a shape similar to barley and are available in different types, including black, red, yellow, and white oats. They are the sixth most produced grain worldwide, following wheat, corn, rice, barley, and sorghum [10,11]. Traditionally, oats have been considered healthy as they are abundant in protein, fiber, vitamins, and minerals and are mainly consumed as oat meals. In particular, oats contain high levels of proteins and lipids as well as balanced amounts of essential amino acids, such as lysine, and 2–6% of β-glucan and are, therefore, recognized as a high-value crop [12,13,14,15]. Compared to other crops that contain an inversely proportional amount of protein to that of lysine, oats consist of a constant amount of lysine regardless of the protein levels [16]. Globulin accounts for 70–80% of the protein content of oats, and they also contain a low amount of prolamin [16,17]. Oats have 5–12% fat, which is higher than that of other cereal crops [18] (Table 1). Approximately 95% of the fat content in oats are palmitic, oleic, and linoleic acids, and 75–80% are unsaturated fatty acids. These unsaturated fatty acids are associated with various beneficial physiological properties, such as the prevention of dementia and antioxidant activity [10,15,19]. Recently, unsaturated fatty acids were shown to lower blood cholesterol, leading to increased interest in oats as a functional food [20,21,22]. Oats also contain polyphenols including caffeic acids, coumaric acids, gallic acids, hydroxybenzoic acids, protocatechuic acids, syringic acids and vanillic acids as the bioactive compounds [2,9,10]. Alkaloids such as avenanthramides (Avns) are also found in oat [9,10]. In addition, oat bran-derived by-products such as proteins, β-glucan, saponin, albumin, prolamins, and glutelins are also valuable for the nutritional components. Therefore, human consumption of whole oat grain is implicated with health benefits due to the acting components in the whole oat grain. Metabolic diseases have been suggested for the benefits and the whole grains or healthy grains has recently been emphasized through systematic meta-analyses of the available information [23].

## 2. Nutritional Benefits of Oats

**Figure 1:**
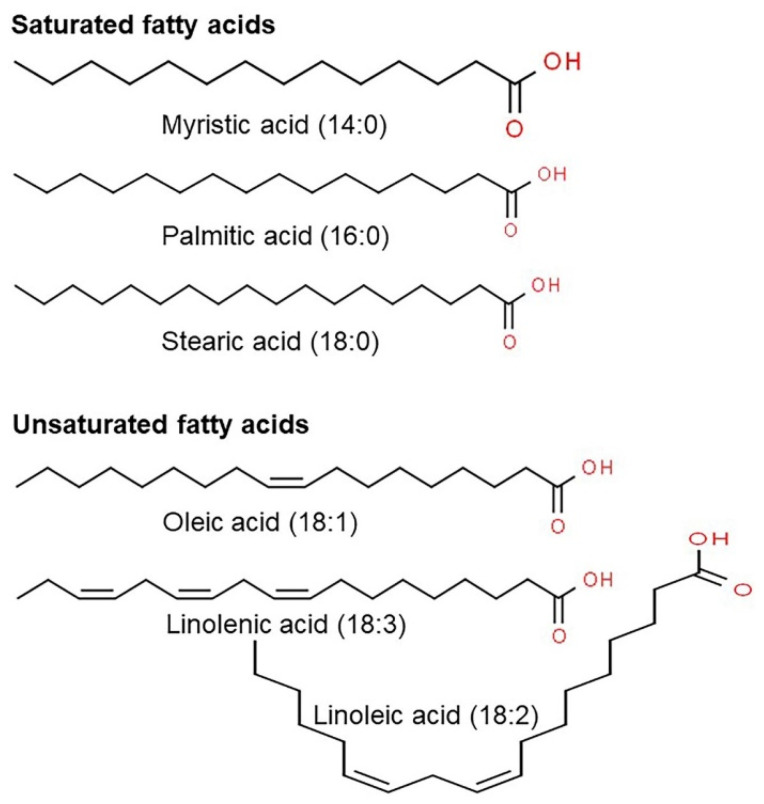
Representative chemical structure of saturated and unsaturated fatty acids present in oats using ChemSpider.

The nutritional benefits of oats as a food product have been studied in different ways. As shown in Table 1, oats are a good source of high-quality proteins, carbohydrates, dietary fibers such as β-glucan and soluble dietary fibers, fat, minerals, phenolic acids, flavonoids, and antioxidants [13,18,24]. Compared to other grains, proteins in oats are superior in quantity and quality, especially for humans and non-ruminants, based on the composition of essential amino acids. Seed storage proteins of grains are divided into globulins, which are soluble in salt, and prolamins, which are soluble in alcohol [16,25]. Among essential amino acids, lysine, which plays a crucial role in protein biosynthesis, is particularly important as this amino acid cannot be synthesized and must be obtained from the external environment in mammals [26]. Grains are a preferred source of these essential amino acids. In particular, proteins in oats are rich in lysine and have a higher ratio of globulin proteins than other grains that have a high prolamin content [27]. Oats generally contain 3–11% of fat, and several strains contain up to 18% of fat. In addition, most of the fats in oats are stored in the endosperm compared to other grains that store a high fat content in the germini and germinal disk [28]. Fatty acids in oats include oleic (18:1), linoleic (18:2), and linolenic acid (18:3), which are unsaturated fatty acids, and myristic (14:0), palmitic (16:0), and stearic acid (18:0), which are saturated fatty acids (Figure 1) [29,30]. Among these, oats contain high amounts of oleic, linoleic, and palmitic acid. Oleic and linoleic acid are nutritionally essential unsaturated fatty acids, and palmitic acid prevents peroxidation of fat, which causes toxicity and reduces the flavor of grains [31,32]. Thus, oats contain nutritionally excellent fat content and fatty acid composition [33]. However, depending on the use of the grain, it is necessary to evaluate the fat content in more detail. The energy value of fat (37 kJ/g) is much higher than that of proteins or carbohydrates (16–17 kJ/g) [34,35]. Thus, grains with high fat content are generally preferred for animal feeds and not for consumption by humans because they lack flavor and brown excessively during cooking [35,36].

## 3. Functionality of β-Glucan in Oats

**Figure 2:**
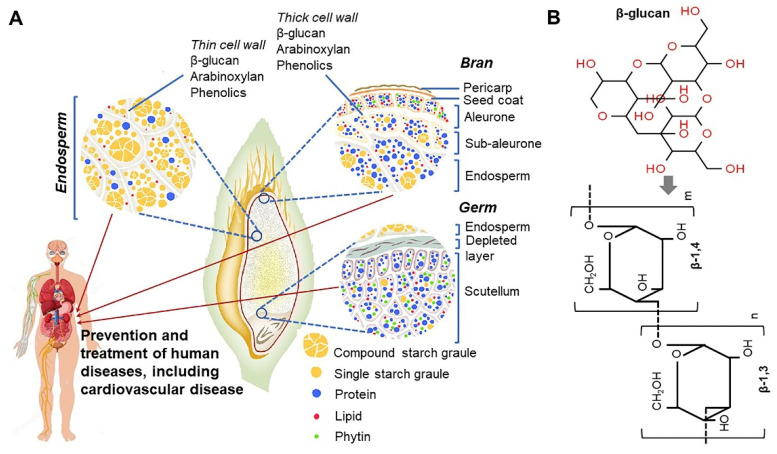
Structural diagram of the seed-derived cellular components from different oat tissue, including bran, germ, and endosperm, and nutrient distribution and organization within these tissues (**A**) [18,40], and chemical structure of β-glucan linked to β-1,3 and β-1,4 bridge (**B**).

**Figure 3:**
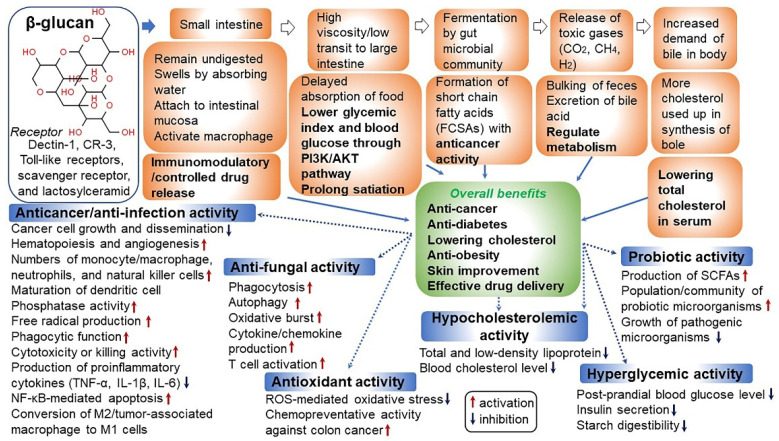
Human health benefits of β-glucan associated with anti-cancer/anti-infection, anti-fungal, antioxidant, hypocholesterolemic, hyperglycemic, and probiotic activity. Binding receptors for β-glucan have been known to be dectin-1, complement receptor 3 (CR3), scavenger receptor, and lactosylceramid. Of these, dectin-1 is a major receptor [18,52,53,54,55].

**Figure 4:**
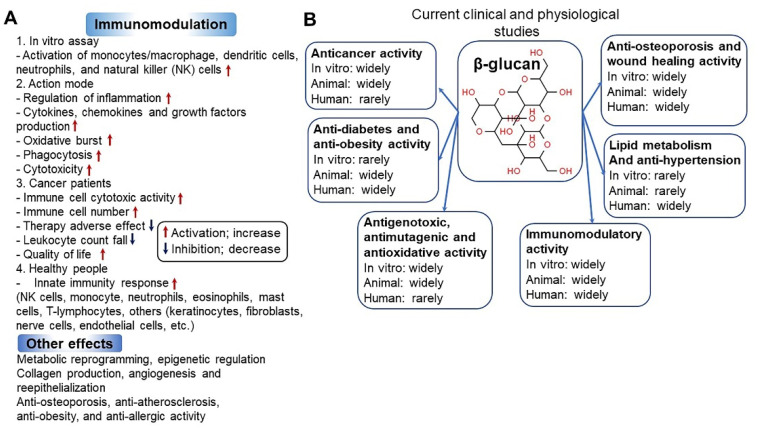
Immunomodulatory activity (**A**) [57] and clinical and physiological perspectives (**B**) [58] of β-glucan.

In 1998, the Food and Agriculture Organization approved the labeling of health effects on food products containing oat extract, and this has attracted attention globally [37]. In addition, oats were selected as one of the world’s top 10 superfoods. Because of the growing interest of consumers in healthy foods, the consumption of oats as an excellent functional food has increased recently [38]. Oats are an important functional ingredient and contain high amounts of dietary fiber and β-glucan. The β-glucan content of oats varies depending on the type and part of the grain (Figure 2A) [39,40]. β-glucans in oats are present as insoluble and soluble dietary fibers. As soluble dietary fibers have superior physiological functions, interest in oats with high soluble β-glucan content has increased. β-glucans in oats mainly consist of (1→3), (1→4)-beta-d-glucan, which is a linear polysaccharide, and this glucan is often abbreviated as β-glucan (Figure 2B) [41,42]. The health benefits of β-glucan in wheat and barley are relatively well-known [43]. β-glucans in oats are known to differ from those in wheat and barley in terms of solubility, gelation, and relative molecular weight [44]. In oats, a large amount of β-glucan is contained in the endosperm and aleurone layer cell wall (Figure 2A) [40]. β-glucan regulates the gastrointestinal transit rate after meals and starch digestion to dilute blood sugar level through changes in the glycemic index, thereby reducing the insulinemic response in diabetes [41,45]. In addition, β-glucan has various physiological effects such as preventing cardiovascular diseases by controlling the blood pressure and anti-obesity and anti-cancer effects (e.g., prevention of colorectal cancer) [18,46,47,48,49,50,51,52,53,54,55]. It lowers low-density lipoprotein (LDL) cholesterol level in the blood and elevates high-density lipoprotein (HDL) cholesterol level to help maintain normal blood lipid concentration and body weight [56]. Furthermore, β-glucan activates leucocytes/macrophages and promotes immune function by increasing immunoglobulin levels, and NK and killer T cell numbers [57,58]. This leads to increased resistance against cancer and infections as well as diseases caused by parasites [57,59,60]. β-glucan water-soluble dietary fiber in oats increases intestinal viscosity and shortens the transit time of the intestinal contents and nutrients during peristalsis [61]. This helps to reduce the absorption rate of proteins, lipids, and glucose, leading to decreased body weight and dietary efficiency (Figure 3 and Figure 4) [40,43,62,63]. The cell wall of oats contains a high level of mixed-linked β-d-glucan that consists of β(1→3) and β(1→4) glucosides in a ratio of 3:7 [64]. This mixed-linked β-d-glucan is also known to lower blood cholesterol levels [56]. β-glucans noted above are a class of fiber identified in yeast, algae, bacteria, fungi, and some plants such as oats and barley [43]. The particulate or soluble form of oat-derived β-glucan appears safe when digested [39]. However, some moderate adverse effects have been reported. Symptoms including ulcerative colitis, diarrhea, back pain, joint pain, kidney disease, circadian disruption-induced metabolic syndrome, bile acid storage and vascular calcium storage are reported to be ameliorated in the specific conditions such as ingestion with a high-dose-limiting concentration of β-glucan [48,49,65,66,67,68]. It remains to be elucidated whether this intake is safe for women who are pregnant or breastfeeding. In addition, dietary intake of β-glucan may be unsafe for an individual with certain disease-related conditions such as acquired immune deficiency syndrome (AIDS) and AIDS-related complex [69]. However, no serious adverse effects related to β-glucan supplementations have been reported during laboratory and clinical trials except in some special cases as mentioned above [49,70,71].

## 4. Fermentation Enhancement of Functionality of Oat β-Glucan and Ingredients

Several previous studies have prepared lactic acid fermented beverages from various plant materials. Soymilk has been extensively studied for its property of improved storage and sensory characteristics after lactic acid fermentation [72]. Oligosaccharides such as raffinose and stachyose, which cause flatulence, are hydrolyzed by lactic acid bacteria containing α-galactosidase, thereby improving the nutritional properties [73]. Lactic acid fermentation of peanut oil reduces the content of *n*-hexanal, a substance that causes a fishy smell in soybean, and thus improves the sensory properties [74]. A study was conducted to assess the changes in the microbiological characteristics to evaluate the growth potential of lactic acid bacteria during fermentation with oat extract as microbial substrate.

Gut microbiome have recently received a light in human health promotion in views of dietary grain consumption and health enhancement. Polyphenolic compounds are rich bioactive components present in grains, indicating the crucial polyphenolic compounds as human health factors [75]. Polyphenolic compounds and fiber present in whole grain diet are helpful to reduce risks of human diseases. Fiber consumption is associated with specific microbial flora such as *Bacteroides.* Dietary fiber such as arabinoxylans, pectins or insulin promotes enrichment of health-related bacterial flora [76]. In gut, the consumed components are known to interact with the microbiome. Therefore, the oat components are suggested to interact with the gut microbiome to exert human health [76]. The dietary consumption of oat fiber and polyphenolic compounds is considered to help gut bacterial promotion associated with healthy intestinal benefit.

It was observed that fermentation using a combination of *Lactobacillus delbrueckii* subsp. *bulgaricus (L. bulgaricus)* and *Streptococcus salivarius* subsp. *thermophilus (S. thermophilus)* increased the number of microorganisms and acid production compared to fermentation using only *L. bulgaricus* or *S. thermophilus*. In addition, fermentation with *S. thermophilus* led to a lower pH than fermentation with *L*. *bulgaricus*. Fermentation using a mix of *L. bulgaricus* and *S. thermophilus* led to a faster decrease in the pH. These results suggest that there is a growth-promoting phenomenon between *L. bulgaricus* and *S. thermophilus*, similar to that in yogurt made from milk. The increased number of microorganisms and acid production and lower pH in fermentation using *S*. *thermophilus* may be attributed to the better adaptation to oat extract of *S*. *thermophilus* than of *L. bulgaricus* [75,76,77,78,79,80]. These studies have demonstrated that the increased content of the β-glucan in oat plantation is associated with maximum health benefits and hence, biosynthetic enhancement of the β-glucan would be an important goal of functional oat breeding.

## 5. Other Bioactive Ingredients and Functionalities

Physiologically active ingredients of oats include vitamin E, carotenoids, anthocyanins, lignans, phytic acid, phenolics, and phytosterol, and Avn, which is a phenol present only in oats [81,82]. These components are secondary metabolites produced as defense mechanisms during plant growth and act as antioxidants that control cell damage from oxidative stress by removing reactive oxygen species in the human body [82,83,84,85]. Furthermore, the addition of oat components during the processing of food products helps to suppress fatty acid plaque development because of its antioxidant action and improves storage properties [23,86,87].

**Figure 5:**
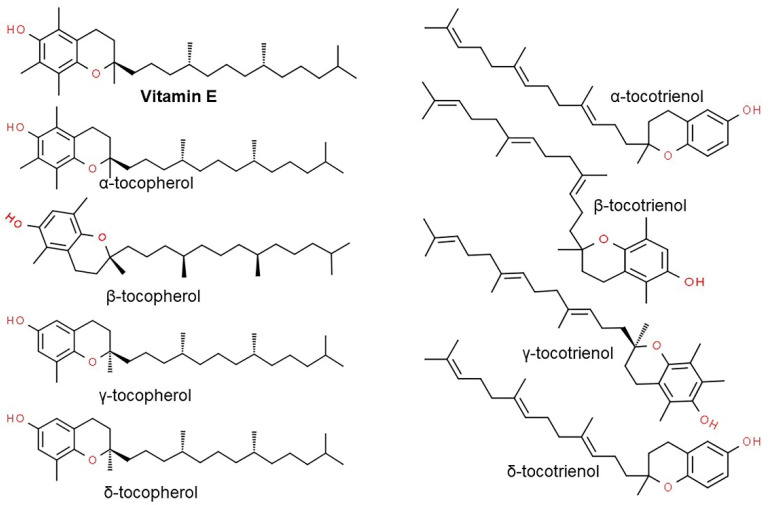
Chemical structure of vitamin E-derived tocopherol and tocotrienol isomers.

**Figure 6:**
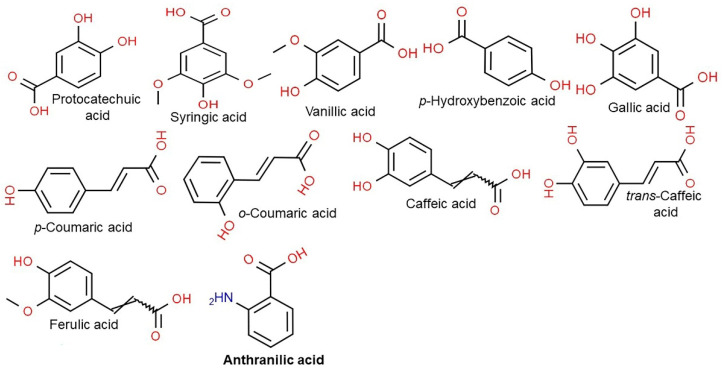
Chemical structure of main polyphenolic compounds found in oats.

**Figure 7:**
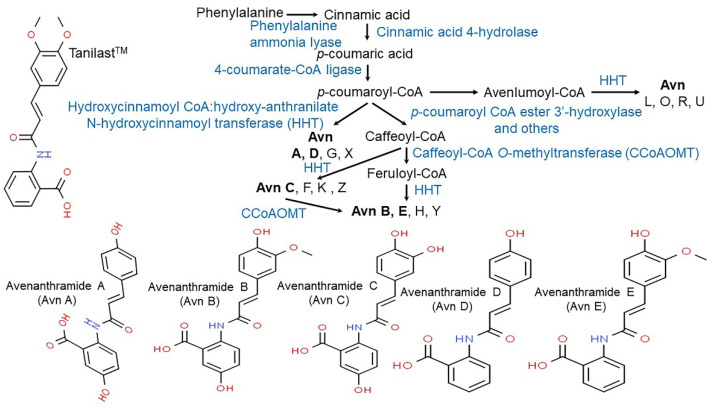
Proposed biosynthetic pathway of major avenanthramides in oat (*Avena sativa* L.) [9,94,95].

**Figure 8:**
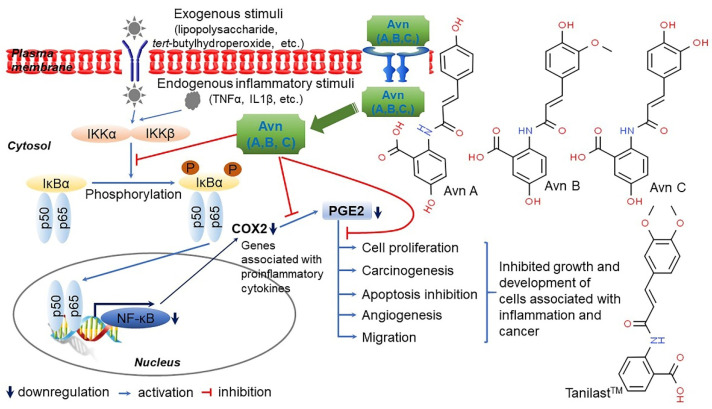
A predicted mechanism of avenanthramides (Avns)-mediated anti-inflammatory in skeletal muscle C2C12 cells. Avns, the polyphenolic molecules identified solely in oats, exhibit anti-inflammatory activity mainly by inducing nuclear factor-kappaB (NF-κB) inactivation in C2C12 cells. Avns downregulated the expression of IκB kinase beta (IKKβ) as an inhibitor of NF-κB kinase subunit beta in cellular response to *tert*-butyl hydroperoxide (tBHP)-meditated oxidative stress and attenuated the expression tumor necrosis factor alpha (TNFα) and interleukin 1β (IL-1β) at the transcriptional level under the same condition. Furthermore, Avns reduced the expression of cyclooxygenase-2 (COX-2) protein, along with decreased prostaglandin E2 (PGE2) levels. The downregulated COX2/PGE2 pathway leads to the inhibition of cell proliferation, migration, apoptosis suppression, angiogenesis, and carcinogenesis in various cell lines. Thus, Avns can be potent inhibitors of NF-κB-mediated inflammatory response following the downregulation of IKKβ activity in C2C12 cells [109,110].

**Figure 9:**
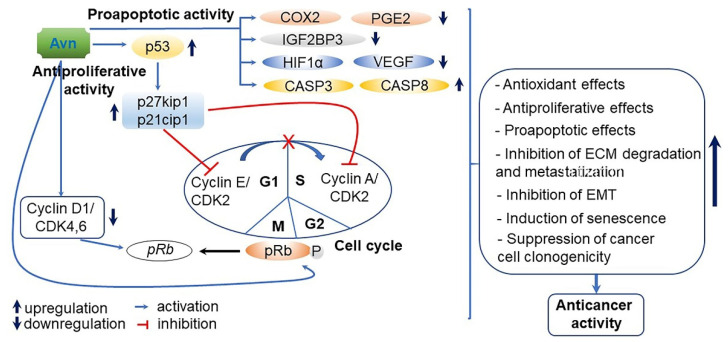
A proposed action mode of avenanthramides (Avns)-derived antiproliferative and proapoptotic activity. p53, and p27kip1 and p21cip1 activated by Avns treatment suppress the expression of cyclin E/ cyclin-dependent kinase 2 (CDK2) and cyclin A/CDK2 associated with cell cycle, and lead to the cell cycle arrest (G1 to S phase). In addition, Avns also downregulate the expression of cyclin D1/CDK4,6 and enhances phosphorylation of Rb protein (pRb) as a tumor suppressor. As a result, Avns cause cell cycle arrest of M phase. Based on these results, Avns play a vital role in the positive control of the cell cycle and in tumor progression [110,111]. With regard to proapoptotic activity, Avns upregulate caspase 3 (CASP3) and caspase (CASP8), while they downregulate insulin-like growth factor 2 mRNA-binding protein 3 (IGF2BP3), hypoxia-inducible factor 1-alpha (HIF1α), vascular endothelial growth factor (VEGF), cyclooxygenase 2 (COX2), and prostaglandin E2 (PGE2) in tumor cell lines [112]. Therefore, Avns reinforce anticancer effects through increased antioxidative, antiproliferative and proapoptotic effects, as well as induction of senescence, and inhibition of extracellular matrix (ECM) degradation and metastatization and epithelial-mesenchymal transition (EMT).

**Figure 10:**
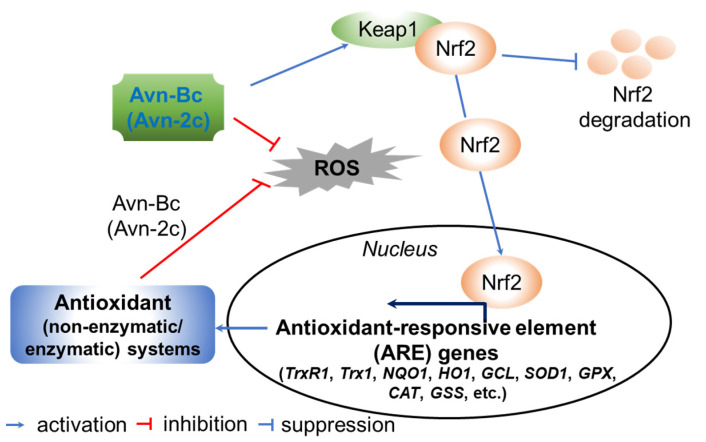
A proposed action mode of avenanthramide (Avn)-derived antioxidant activity. Avn-Bc unties the complex of nuclear factor erythroid 2-related factor 2 (Nrf2) and Kelch-like ECH-associated protein 1 (Keap1). Detached Ntf2 moves to the nucleus, binds to the antioxidant-responsive element (ARE) domain, and activates a wide range of cytoprotective and antioxidative genes, including thioredoxin reductase (TrxR1), thioredoxin 1 (Trx1), NAD(P)H:quinone oxidoreductase (NQO1), heme oxygenase 1 (HO-1), glutamate-cysteine ligase (GCL), superoxide dismutase 1 (SOD1), glutathione peroxidase (GPX), catalase (CAT) and glutathione synthetase (GSS), at the transcriptional and translational levels under oxidative stress conditions. The activated antioxidant systems improve redox homeostasis by neutralizing reactive oxygen species (ROS). Thus, Avn-Bc enhances cell homeostasis in response to oxidative stress [99,112].

**Figure 11:**
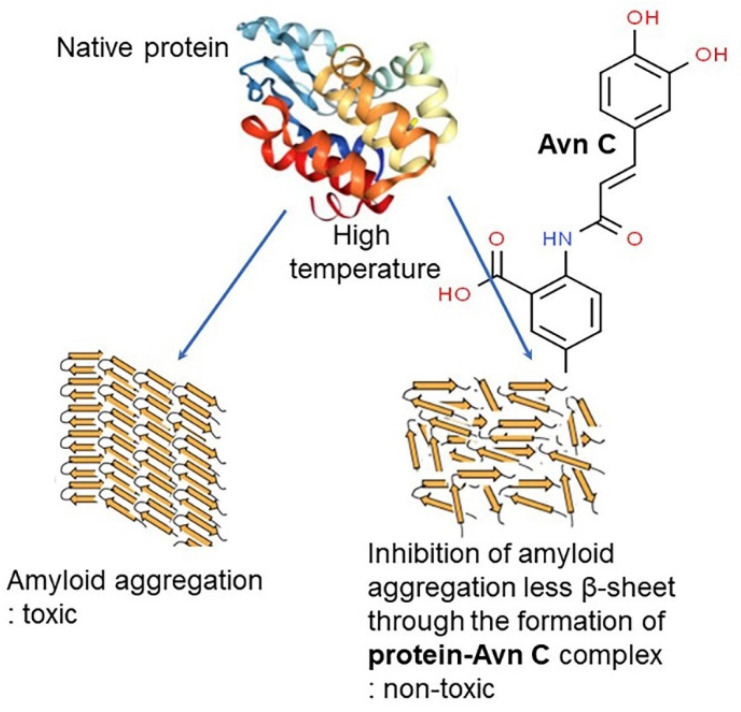
Prevention of amyloid formation in the presence of avenanthramide C (Avn C). The assembly of misfolded protein into amyloid fibrils that have a high β-sheet-rich secondary structure is associated with many human diseases, including central nervous diseases (Parkinson’s, Alzheimer’s, and Huntington’s disease), amyotrophic lateral sclerosis, and type 2 diabetes, and diseases related to the accumulation of insoluble serum amyloid A protein in liver, spleen, and kidney. Although great efforts have been made to elucidate the pathogenesis of these diseases and development of effective therapy to date, there is still no evidence for the treatment and prevention associated with amyloid-related diseases. Polyphenols such as avenanthramides have been widely studied as a key factor of amyloid aggregation inhibitors. Their bioactive effects depend on the number and position of hydroxyl groups around the flavone backbone. Avn C can act as a potential biomolecule in inhibiting protein aggregation by decreasing the formation of β-sheet structure of protein aggregates [115].

Vitamin E consists of four tocopherol isomers (α-, β-, γ-, and δ-tocopherol) and four tocotrienol isoforms (α-, β-, γ-, and δ-tocotrienol) (Figure 5) [88]. Among these, α-tocotrienol has 40–60 times greater antioxidant capacity than β-tocotrienol, a key antioxidant [89]. It lowers blood cholesterol, has anti-inflammatory effects, and inhibits tumor cell proliferation in humans [90]. The main polyphenolic compound found in oats includes protocatechuic, syringic, vanillic, *p*-hydroxybenzoic, gallic, *p*-coumaric, *o*-coumaric, and caffeic acids (Figure 6) [91,92,93]. Among them, Avn biosynthesized from phenylalnine as an alkaloid (Figure 7) [9,94,95] is a polyphenol with various physiological properties, including antioxidant, anti-inflammatory, anti-cancer, anti-thrombotic, anti-proliferative, and anti-itch activities [9,95,96,97,98,99,100,101,102,103]. Avn has 30 times higher antioxidant activity than other phenolic compounds [98,99,102]. There are various types of Avn found in oats. Depending on the residue of N-cinnamoyl anthranilic acid, Avn A combined with *p*-coumaric acid, Avn B combined with ferulic acid, and Avn C combined with caffeic acid are mainly found in oats [101,102,104]. The structure of Avn is similar to tranilast, a commercially available anti-allergic drug, and many studies assessed the anti-inflammatory and anti-atherogenic effects of Avn [105,106,107,108]. In particular, Avn is known to inhibit the release of inflammatory substances by macrophages or adhesion of monocytes to vascular endothelial cells and exhibit anti-cancer effects through anti-proliferative and pro-apoptotic activities (Figure 8, Figure 9 and Figure 10) [78,99,106,107,108,109,110,111,112,113]. In a recent study, Avn C, among different types of Avn, was shown to be effective against dementia and hearing loss [98,114], as well as prevention of misfolded aggregation (Figure 11) [115]. Avn is also helpful in relieving itching of dry skin, and oat extract is widely used as a cosmetic material in Western Europe [65]. Avns (A, B and C) dose-dependently inhibit cellular tyrosinase and melanin synthesizing tyrosinase activities with the competitive inhibitory manner. They also inhibit the expression level of melanogenic proteins (TRP1 and 2). The tyrosinase-binding affinity of Avns, which obtained by molecular docking simulation and the Derek Nexus quantitative structure–activity relationship system, suggests that the binding affinities are ranged between −7.5 kcal/mol and −6.8 kcal/mol [116]. Moreover, creams containing oat extract showed improved facial skin and were safe to use [117]. Based on these findings, it is possible to explore the feasibility of oat extract as an active ingredient in functional cosmetics to alleviate redness and pigmentation. In addition, combining oats with other natural ingredient extracts for cosmetic products to cover wrinkles, whiten, alleviate redness, and have other functions can lead to several positive changes [65].

**Figure 12:**
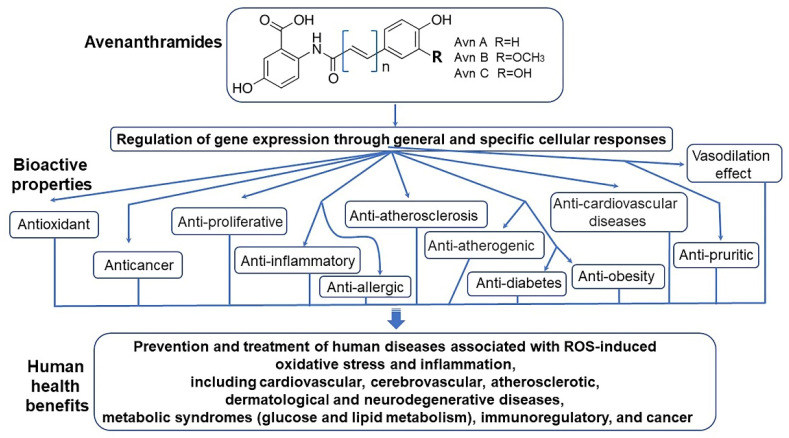
Physiological bioactivities of oat-derived avenanthramides [65,110,120].

In a study that compared the antioxidant effects and inhibition of cancer cell proliferation of oat extract according to extraction solvents, the antioxidant activity of the extract was measured by assessing the scavenging of 2,2´-azinobis 3-ethylbenzothiazoline-6-sulphonic acid (ABTS) and 1,1-diphenyl-1-picrylhydrazyl (DPPH) radicals and reducing power, whereas the inhibition of cancer cell proliferation was assessed using colorectal, lung, and breast cancer cell lines. It was observed that the total polyphenol content, scavenging of ABTS and DPPH radicals, and reducing power were the greatest in methanol extracts [118]. In addition, methanol extracts had the most significant inhibitory effects on the proliferation of colorectal cancer (HCT116), lung cancer (NCI-H460), and breast cancer (MCF7) cells [119]. Although there were differences in the antioxidant effects and inhibition of cancer cell proliferation of oats depending on the extraction solvent, these findings demonstrate that oats have antioxidant and anti-cancer effects [98,120]. Notably, to date no studies have shown any adverse effects associated with Avn supplementation. Altogether, physiological activities of oat-derived Avns were summarized into Figure 12 [65,110,121].

## 6. Functionality of Sprouted Oats

In light of the nutritional value of ordinary oats, interest in sprouted oats is also gradually increasing. Germination is a process in which the seed absorbs moisture and undergoes various metabolic processes to produce young roots and shoots. The germinal disk with determined genetic information germinates under suitable conditions, and biological processes are induced by activities of enzymes that decompose starch, resulting in the generation of a new plant [96,122]. During germination, various enzymes, nutrients, and genetic information in the germinal disk and endosperm of the seed are activated, and the maximum amount of nutrients is secured. In detail, the germinal disk germinates, and proteins undergo qualitative changes. Amino acids, carbohydrates, fatty acids, and vitamins B1, B2, and E are increased, and minerals and dietary fibers are changed (Figure 2). In addition, physiologically contents of active ingredients such as γ-aminobutyric acid, γ-oryzanol, and arabinoxylan are enhanced [123,124,125]. During germination, enzymes are activated, and softening of the grain can lead to improved texture. In addition, the digestibility of carbohydrates increases, leading to increased absorption of nutrients in the body. Moreover, germination extensively changes the chemical composition of grains [126,127,128]. For example, soaking barley in water and germinating them increases the biodegradability of proteins, vitamins, minerals, and other substances in barley, leading to improved physiological activities [129]. In rye, phytosterol, folate, lignan, and phenolic contents increase during germination [130]. Likewise, a similar phenomenon is observed in oats. The concentration of Avn in oats is significantly increased after germination compared to that before germination [9,131,132]. Furthermore, germination of grains can lead to saccharification effects, which can improve palatability such as by enhancing flavor [126,133]. Thus, various types of processed foods and functional products can be manufactured using these germination characteristics.

## 7. Functional Enhancement Using Oat By-Products

**Figure 13:**
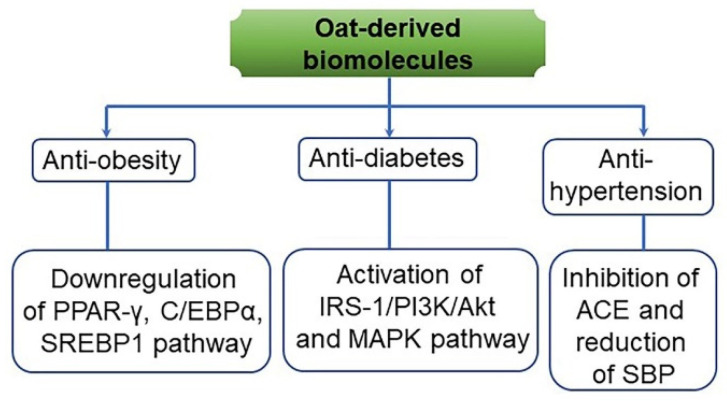
A predicted action mode of anti-obesity, anti-diabetes, and anti-hypertension effects from oat-derived biomolecules and by-products. Anti-obesity is associated with the downregulation of peroxisome proliferator-activated receptor gamma (PPAR-γ), CCAAT-enhancer-binding protein isoform alpha (C/EBPα), and sterol regulatory element-binding protein 1 (SREBP1). Anti-diabetes is involved in the activation of insulin receptor substrate 1 (IRS-1)/phosphoinositide 3-kinase (PI3K)/protein kinase B (Akt) and AMP-activated protein kinase (AMPK) signaling pathway. Anti-hypertension is related to the inhibition of angiotensin-converting enzyme (ACE) and the reduction of systolic blood pressure (SBP) [147].

Recently, studies on the creation of new value-added products from food by-products have been actively conducted [134]. Thus, a considerable amount of by-products such as husks, seeds, grains, and bran are thrown away as wastes [135]. Although food by-products have been mainly used as animal feed until now, some of these by-products can be used in eco-friendly industries [135,136,137]. In addition, these by-products such as sugar cane, fruit beets, whey, bread, and wheat by-products as natural materials are also utilized for functional components [55,138,139,140]. However, the functional properties and nutritional value of these by-products have not been actively studied, although by-products can be utilized [141]. Food by-products are considered high-value food additives for their antioxidant, anti-bacterial, colorant, and flavoring functions [142,143,144,145]. More than 20 million tons of oats are annually produced, mainly as food or animal feed [146]. Oat bran is a key by-product and accounts for approximately 50% of the weight of dry oat grains [147]. Oat bran also contains high-quality proteins such as β-glucan, saponin, albumin, prolamins, and glutelins that lower cholesterol levels (Figure 13) [148,149,150,151,152]. To date, studies on oats mainly assessed the nutritional value and processing characteristics of oat grains or powder [13,42,55]. There is a lack of studies on its by-products, such as the bran, hull, and leaves, which are mostly thrown away as waste during the processing [153]. However, as recent studies reported the content of numerous functional components in these by-products, the utility of oats is gradually increasing [42,55].

## 8. Importance of Oat Breeding

**Figure 14:**
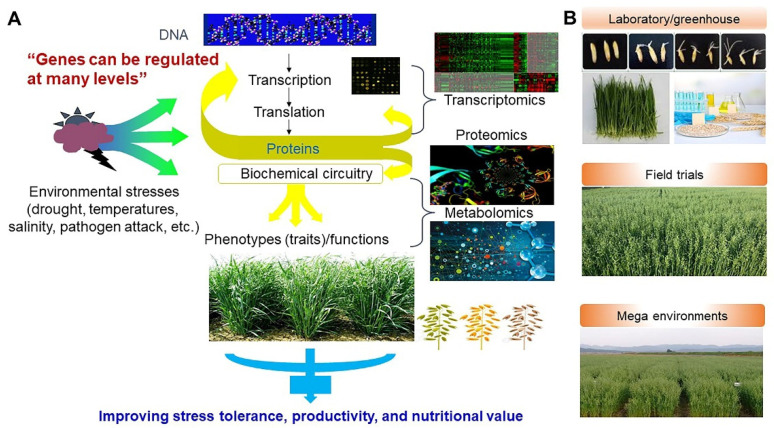
Breeding strategy of oat crop plants to improve stress tolerance, productivity, and high nutritional value based on transcriptomics, proteomics, and metabolomics (**A**), and evaluation of agronomic traits on laboratory and greenhouse conditions, field trials, and mega environments (**B**).

Compared to other grains, oats grow well in harsh environmental conditions with insufficient nutrients, can be cultivated in cold and humid climates, have low chemical fertilizer requirements, have fewer pests, and require less chemical control, making them eco-friendly [154]. Approximately 70% of the produced oat grains are consumed for animal food, and the remaining 30% are consumed as food products for humans [155]. However, farmers prefer crops such as wheat, rice, and barley, which provide higher yields and profits compared to oats, and thus, oat production has gradually decreased over the past few decades globally. As a result, unlike wheat, barley, and rice, agricultural research on oats is limited [156]. Previous studies by oat breeders mainly focused on phenotype selection and disease resistance related to the yield, and there are fewer studies on genetics and other necessary traits of oats than those of other grains [157,158,159]. However, following the recognition of their excellent nutritional effects and health benefits, studies on oats and their consumption have steadily increased, including in Korea [24]. However, studies have been mainly focused on the cultivation or quality evaluation as fodder crops and development of bulky feed varieties rather than edible varieties [160,161,162]. Moreover, previous studies often assessed the extraction and physiological activity of its functional component, β-glucan [163]. Thus, the development of new varieties for food or processed products has not been actively conducted. In particular, studies on genetic resources as breeding materials and breeding for improved nutritional function are limited [164]. However, the public awareness regarding the health functionalities of oats has rapidly enhanced their consumption, and such improvement in nutritional function may have positive effects in promoting their utilization in the future [165,166]. Therefore, future studies should not only actively seek to increase the yield but also improve the nutritional value by cultivating varieties with high contents of Avn and β-glucan (Figure 14) [23,166,167,168,169].

## 9. Conclusions and Perspectives

Oats, which belong to the Poaceae family, grow well in cool and humid weather and are annual crops. Oats are aware of the surrounding environment with superior senses compared to other plants, effectively compete with limited resources available in the soil and atmosphere, and accurately judge the surroundings. Oats also have the ability to determine and implement strategies to respond to environmental stimuli. In other words, oats detect changes and produce various useful substances while being sensitive to altered environmental conditions. The physiological activities of oats, such as lowering blood cholesterol levels, have been demonstrated in studies, and thus, interest in oats as a functional food has increased. Oats contain 7–14% of dietary fiber and 3–5% of β-glucan, which is one of the important functional components of oats. β-glucan is a water-soluble dietary fiber found in oats and exhibits viscosity. It can reduce the risk of heart disease by inhibiting the absorption of cholesterol in the intestine. β-glucan exhibits anti-obesity effects by inhibiting the accumulation of adipocytes (thereby reducing the formation of body fat), preventing the accumulation of cholesterol in the liver, and improving lipid metabolism. Furthermore, Avn, which is present only in oats, has various physiological activities along with β-glucan in increasing the nutritional and functional values of oats. The oat β-glucans are potentially suggested as a celiac disease-targeting diet resource, especially for gluten-free diets [165]. However, the scientific studies to apply the β-glucans for the autoimmune diseases are not performed yet. Oats also contain high levels of tocotrienol (another type of vitamin E), which possesses antioxidant and anti-cancer effects and improves hyperlipidemia. Therefore, oats embody a much more resistant and modern crop model than other plants.

Oats are a prime example of a combination of solidity and flexibility. The modular composition of oats is the essence of modernization, and oats can actively respond to repeated changes in the environment without losing their functions through their distributed cooperative structure without a control center for quick adaptation. Their unique evolution has led to solutions different from those of other plants, suggesting that oats are much more modern than other plants. Thus, the unique characteristics of oats may offer several opportunities for the development of novel functional foods in the coming years.

Oats are important to Koreans, who mainly consume rice. Many Koreans reduce the proportion of rice in meals to decrease sugar intake; therefore, oats, which have a good nutritional balance, are an excellent alternative to rice or can be mixed with rice. Asians, who consume grain-oriented meals, often lack essential amino acids that must be additionally obtained from protein foods. Thus, the intake of oats can help to improve the consumption of more high-quality proteins such as soybeans. In addition, individuals often suffer from low immunity due to stress, overwork, excessive drinking, chronic fatigue, lack of sleep, and incorrect dietary habits. Although they are aware of their problems, proper health management in a busy life is not easy, leading to the intake of expensive nutritional supplements or health foods. However, it is necessary to balance the nutrients in daily meals naturally. The intake of essential nutrients leads to improved immunity to fight against various stresses. Oats, containing various nutrients in small amounts, would help prevent and treat different adult diseases by enhancing the immune system through nutritional balance relatively easily. Therefore, future studies on oats must not only actively seek to increase the yield but also improve the nutritional value by cultivating varieties with high contents of Avn and β-glucan. In addition, studies should simultaneously assess the physiological activity and related mechanisms of functional ingredients.

## Figures and Tables

**Table 1:** Comparison in the functional components in white rice, wheat, oat, and barley [18].

Components	Oat (%)	Barley (%)	Wheat (%)	White Rice (%)
Protein	9–17	14.2	7–22	6.3
Fat	5–12	2.4	~2.5	0.7
Starch	27–50	54.2	68	80.1
Total dietary fiber	13–30	13.1	11.5–15.5	1
β-glucan content in grain (g/100 g)				
	3–8	2–20	0.5–1	0.13
